# Employing Demand-Based Volumetric Forecasting to Identify Potential for and Roles of Devices in Scale-Up of Medical Male Circumcision in Zambia and Zimbabwe

**DOI:** 10.1097/QAI.0000000000000991

**Published:** 2016-05-24

**Authors:** Francine Fram, Fred Church, Maaya Sundaram, Sema K. Sgaier, Renee Ridzon, Maria Eletskaya, Alice Nanga, Sehlulekile Gumede-Moyo, Bushimbwa Tambatamba, Owen Mugurungi, Getrude Ncube, Sinokuthemba Xabayu, Patrick Odawo, Steve Kretschmer

**Affiliations:** * Ipsos Healthcare, London, United Kingdom;; † Integrated Delivery, Global Development Program, Bill & Melinda Gates Foundation, Seattle, WA;; ‡ Department of Global Health, University of Washington, Seattle, WA;; § Surgo Foundation, Seattle, WA;; ‖ Department of Global Health and Population, Harvard T. H. Chan School of Public Health, Boston, MA;; ¶ Department of Epidemiology, Boston University School of Public Health, Boston, MA;; # Ministry of Community Development, Mother and Child Health, Lusaka, Zambia; and; ** Ministry of Health and Child Care, Harare, Zimbabwe.

**Keywords:** voluntary medical male circumcision, circumcision, demand generation, devices, forecast, market research

## Abstract

**Introduction:**

Devices for male circumcision (MC) are becoming available in 14 priority countries where MC is being implemented for HIV prevention. Understanding potential impact on demand for services is one important programmatic consideration because countries determine whether to scale up devices within MC programs.

**Methods:**

A population-based survey measuring willingness to undergo MC, assuming availability of surgical MC and 3 devices, was conducted among 1250 uncircumcised men, ages 10–49 years in Zambia and 1000 uncircumcised men, ages 13–49 years in Zimbabwe. Simulated Test Market methodology was used to estimate incremental MC demand and the extent to which devices might be preferred over surgery, assuming availability of: surgical MC in both countries; the devices PrePex, ShangRing, and Unicirc in Zambia; and PrePex in Zimbabwe.

**Results:**

Modeled estimates indicate PrePex has the potential to provide an overall increase in MC demand ranging from an estimated 13%–50%, depending on country and WHO prequalification ages, replacing 11%–41% of surgical procedures. In Zambia, ShangRing could provide 8% overall increase, replacing 45% of surgical procedures, and Unicirc could provide 30% overall increase, replacing 85% of surgical procedures.

**Conclusions:**

In both countries, devices have potential to increase overall demand for MC, assuming wide scale awareness and availability of circumcision by the devices. With consideration for age and country, PrePex may provide the greatest potential increase in demand, followed by Unicirc (measured in Zambia only) and ShangRing (also Zambia only). These results inform one program dimension for decision making on potential device introduction strategies; however, they must be considered within the broader programmatic context.

## INTRODUCTION

The efficacy of medical male circumcision (MC) in the reduction of female-to-male HIV transmission has been demonstrated by observational studies and randomized controlled trials, and MC is recommended for HIV prevention in countries with high HIV prevalence and low MC prevalence.^1–5^ Although MC programs have been scaling up, the current uptake of MC is too low to reach the goal of 80% MC prevalence within the established time frame.

Innovative solutions to simplify the procedure endeavor to increase acceptability and appeal and decrease implementation difficulties.^6^ WHO prequalification has been granted for 2 devices for adolescent and adult circumcision, PrePex and ShangRing.^7,8^ A third, Unicirc, is in clinical development. PrePex is an elastic collar compression device, consisting of a rigid inner ring and an elastic outer ring that restrict blood flow resulting in ischemia and necrosis of the foreskin; it does not require a sterile field or injected anesthesia. ShangRing is a collar clamp device, consisting of 2 concentric plastic rings that provide tight compression to achieve hemostasis, at which time the live foreskin tissue is removed. A sterile field and injected anesthesia are required. For both of these devices, a follow-up visit is required for device removal and healing is 1–2 weeks longer than for surgery.^9^ Despite this, both have been shown to achieve higher client satisfaction from a cosmetic point of view compared with surgery.^10,11^ Unicirc is a clamp that applies tight compression to achieve hemostasis and tissue sealing. After excision of the foreskin, tissue adhesive is used to close the wound, and the device is removed at the same visit, thus no follow-up visit is required for removal. A sterile field is required; however, the procedure does not require injected anesthesia.^12^ In trials, medical eligibility and rates of successful circumcision were adequate for both PrePex and ShangRing (sufficient data for Unicirc are not available). Rates of adverse events for PrePex and ShangRing are comparable to surgery.^13,14^ All 3 devices require a shorter procedure time than surgery (including application and removal) and no sutures.

The incorporation of these devices into MC programs may lead to increases in MC uptake and/or replacement of the existing surgical service. Incremental demand is defined as the increase in circumcisions performed if a device was available in addition to surgery that could extend circumcision to those who would otherwise remain uncircumcised. Replacement is defined as the number of circumcisions performed with a device that would otherwise have been performed surgically, if only surgery were available. Replacement may be beneficial if device-based methods are easier or more affordable and harmful if cost savings and/or service improvements are negligible and insufficient to offset device training and logistical costs. Estimating the potential incremental demand and/or replacement by devices is essential to inform decisions on device implementation.

MC is part of the national HIV prevention strategy in Zambia and Zimbabwe.^15,16^ In 2014, we conducted primary research to assess whether device incorporation into MC programs would generate incremental demand and the extent to which devices could replace surgery. Despite increasing levels of circumcision in both countries, resulting in 32.6% of the country goal in Zambia and 10.6% in Zimbabwe by 2013,^17^ a dramatic increase in scale-up of MC is needed. Several supply-side factors, such as shortages of trained providers, infrastructure, and transportation capacity for outreach, contribute to low target achievement.^18^ Another critical contributing factor is low demand for MC.^19^ Although there is evidence on the safety and acceptability of devices, and the potential of devices to increase efficiencies,^20^ there are no systematic primary data on client preferences and whether alternatives in circumcision methods could increase demand.

## METHODS

### Study Sample and Data Collection

Data were collected through a structured, quantitative survey in each country in 2014. Survey samples consisted of uncircumcised men in the age range aligned to the national policy for MC. Districts in each country with the most uncircumcised men were chosen, cumulatively accounting for 80% of the goal defined by the national MC strategies, (38 of 72 districts in Zambia, and 35 of 61 districts in Zimbabwe). Samples were distributed by age in proportion to the population size for each age group in each district. In selected districts, households were randomly sampled and 1 man per household was approached. If more than 1 eligible man resided in the household, selection was made through using a preassigned table of random numbers.^21^ Once a quota for an age group was reached in a district, only men who met open quota criteria were interviewed. If the household's selected man was not available or ineligible, the next household was approached. Refusal rates (4.7% in Zambia and 4.9% in Zimbabwe) were low and in line with similar research and most were due to not having time to participate. Participants were interviewed face-to-face and a mobile phone-based data collection platform was used to automate questionnaire logic, MC procedure profile randomization, sample quota management, and data collection.

**FIGURE 1.**
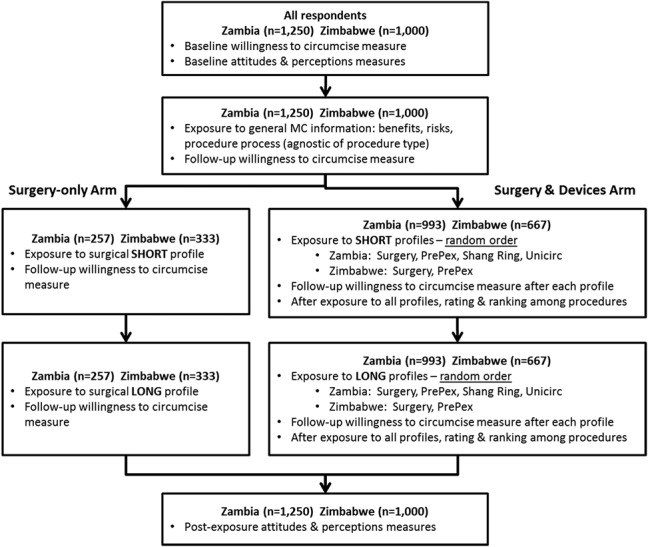
MC device forecast questionnaire consort flow diagram.

In each country, the sample was split into 2 arms. The Surgery-only Arm (n = 257 in Zambia, n = 333 in Zimbabwe) provided an estimation of the potential increase in MC demand resulting from receiving complete information provided on the existing surgical service, unbiased by exposure to device procedure profiles. The Surgery and Devices Arm (n = 993 in Zambia, n = 667 in Zimbabwe) provided an estimation of the potential increase in MC demand and/or replacement generated by availability of devices as alternative procedure to surgery (Fig. 1).

For each arm, the questionnaire included 5 sequential sections: (1) ratings of agreement with statements describing attitudes and perceptions about MC using a 7-point scale and measurement of willingness to undergo MC using a 5-point scale for baseline and each subsequent postexposure measure of willingness to be circumcised; (2) exposure to general MC information, benefits, risks and procedure process (agnostic of procedure type), and postexposure willingness to undergo MC; (3) exposure to “short” profile(s) for procedure type (surgery only in the Surgery-only Arm or randomized order of surgery and each device profile in the Surgery and Devices Arm) and postexposure willingness to be circumcised, including rating each of the procedures after exposure to all procedure short profiles; (4) exposure to “long” profile(s) and postexposure willingness to be circumcised (as outlined above for the short profiles); and, (5) postexposure ratings of agreement with statements describing attitudes and perceptions about circumcision.

The general information provided on MC was reflective of the information an interpersonal communicator would share, agnostic of procedure type. The “short” profiles were reflective of information an interpersonal communicator would share about each MC procedure type and measures of postexposure willingness to be circumcised were used to estimate the proportion of men going to clinics to receive MC through surgery or one of the devices. The “long” profiles reflected information given in a clinic counseling session before circumcision and measures of postexposure willingness to be circumcised were used to estimate switching of preferred circumcision methods “at the clinic”, once full counseling information is provided.

The profiles for surgery and PrePex were developed from existing materials in use by MC programs. Because ShangRing and Unicirc are not yet routinely in use, the profiles were developed in consultation with stakeholders experienced using these devices and development of demand and counseling materials. With all 3 devices, profiles assumed use of topical rather than injected anesthesia. Although the WHO prequalification for ShangRing requires injectable anesthesia, the profile used topical anesthesia because this is being used successfully with ShangRing in China and is being studied in Kenya (personal oral communication, January 2016, Mark Barone, DVM).

Zambian participants viewed profiles for surgery, as well as the PrePex, ShangRing, and Unicirc procedures. Participants in Zimbabwe viewed profiles for surgery and PrePex. Eligible boys younger than 18 years were interviewed with their parents/guardians for ethical reasons and to capture the “decision dynamic” between the boy and parent/guardian. All parents/guardians provided informed consent and boys assented to participate.

### Analytical Approach

**FIGURE 2.**
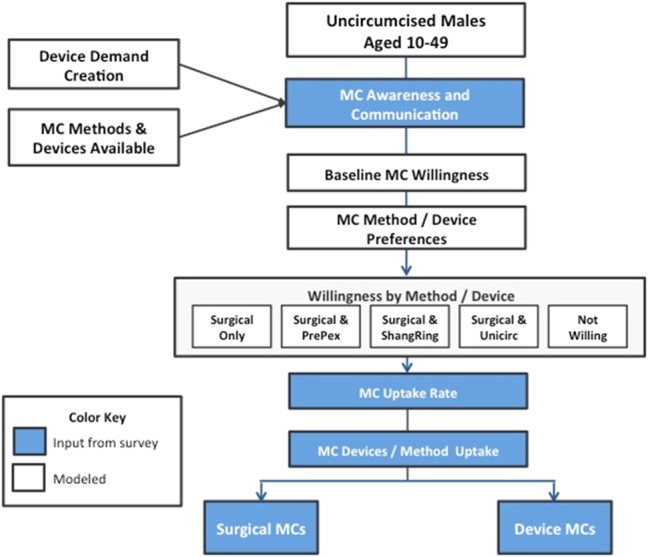
The MC STM model.

A simulated test market (STM) model (Fig. 2) was used to measure incremental demand generated by complete information for surgery and by PrePex, ShangRing, and Unicirc devices (by country), as well as the extent of replacement of surgery by each device. The model was designed to simulate the MC experience, from becoming aware of MC as a way to prevent HIV and other infections to the final outcome of completing the procedure.

STM models were developed in the 1970s to provide information about new products or services, measure willingness to purchase products or services after exposure(s) to information and accurately convert consumer responses into purchase probabilities. STM models have been used to forecast new product demand for consumer packaged goods, pharmaceuticals, and medical devices. In developed markets, STM techniques report validation of ±10% of the forecast 90% of the time.^22,23^ In developing markets, it is not possible to quantify accuracy/uncertainty because the assumptions are based on the market researchers' experience-informed opinions, and CIs around point estimates cannot be provided.

The STM model to measure demand relies on “willingness to be circumcised” questions to determine uptake. Respondents used a five-point scale to answer: considering what you have just seen, which statement best describes how willing you would be to undergo circumcision using this procedure, if the service would be free to you? *Definitely Would*, *Probably Would*, *May or May Not*, *Probably Would Not*, and *Definitely Would Not*.^24,25^

STM models remove respondent overstatement by applying lessons from studies comparing stated and actual consumer behavior and through mathematical modeling. Overstatement factors were developed from longitudinal studies comparing responses to actual uptake for products and medical treatments and from analysis of country program MC data. Weighted overstatement factors were applied to determine the probability of actual uptake if the MC procedure in question were available.

To limit order bias where multiple profiles are tested, only the first and second exposures to profiles are used to estimate demand. Responses from participants, who saw surgery followed by PrePex, or PrePex followed by surgery, are used to estimate surgery and PrePex uptake for scenarios where surgery and PrePex are the only available circumcision methods. Similarly, only response data for respondents who saw surgery followed by ShangRing, or ShangRing followed by surgery, as the first and second profiles are used to estimate surgery and ShangRing uptake for scenarios where surgery and ShangRing are the only available circumcision methods, etc. The first- and second-order sample sizes for each procedure combination in each country were as follows: Zambia—surgery and PrePex (n = 162), surgery and ShangRing (n = 170), and surgery and Unicirc (n = 172); Zimbabwe—surgery and PrePex (n= 666).

MC uptake in each country during 2014 was used for baseline MC volumes. Potential incremental demand and/or replacement were estimated for: (1) complete information for surgery-only (Zambia and Zimbabwe); (2) surgery + PrePex (Zambia and Zimbabwe); (3) surgery + ShangRing (Zambia); and, (4) surgery + Unicirc (Zambia). Awareness of the benefits and risks for surgery and each device and availability of surgery, PrePex, ShangRing, and Unicirc were assumed to be equivalent to that for surgery in 2014. The proportion of aware, motivated men considering MC within the target populations in Zambia and Zimbabwe was derived from the level of awareness determined by questionnaire responses. The medical eligibility rate for Unicirc and ShangRing was assumed to be a 99%; for PrePex it was assumed to be 90% for men aged 18+, and 64% for boys aged 13–17.^26^ Although they were included in the survey sample for Zambia, projections of incremental demand for all MC devices excluded boys aged 10–12 due to the lack of data on medical eligibility and potential WHO prequalification for this age group.

Potential barriers to MC service delivery, such as introduction, distribution, stocking, availability of trained providers, and coordination of services with demand were assumed to be equivalent to levels for surgical MC in 2014. Although modeling the effect of a gradual scale up of demand generation and supply on device uptake would provide a more realistic assessment of the impact of devices on MC demand, this approach was not completed because plans and support for the introduction and integration of devices were unknown.

## RESULTS

### Potential for Devices in Scale-Up of MC, Zambia

In Zambia, providing complete information on the surgical procedure could provide an estimated 5% increase in demand for MCs (Table 1). With awareness and service availability equal to surgery in 2014, PrePex could have increased the total number of MCs by 23% for those aged 18–49. Of the total surgical procedures performed in 2014, our model estimates that 20% would have been replaced using PrePex. With WHO prequalification of PrePex for 13–17 year olds, modeling suggests it could have produced an estimated 41% incremental demand for MCs, while replacing an estimated 36% of existing surgical procedures. Assuming WHO prequalification for 13–17 year olds and wide availability in 2014, Unicirc could have created a 30% incremental increase, with 85% replacement, whereas ShangRing could have created an 8% increase, with 45% replacement of surgeries. Preferences among the 4 procedure types did not vary significantly by age group (Table 3).

**TABLE 1.**
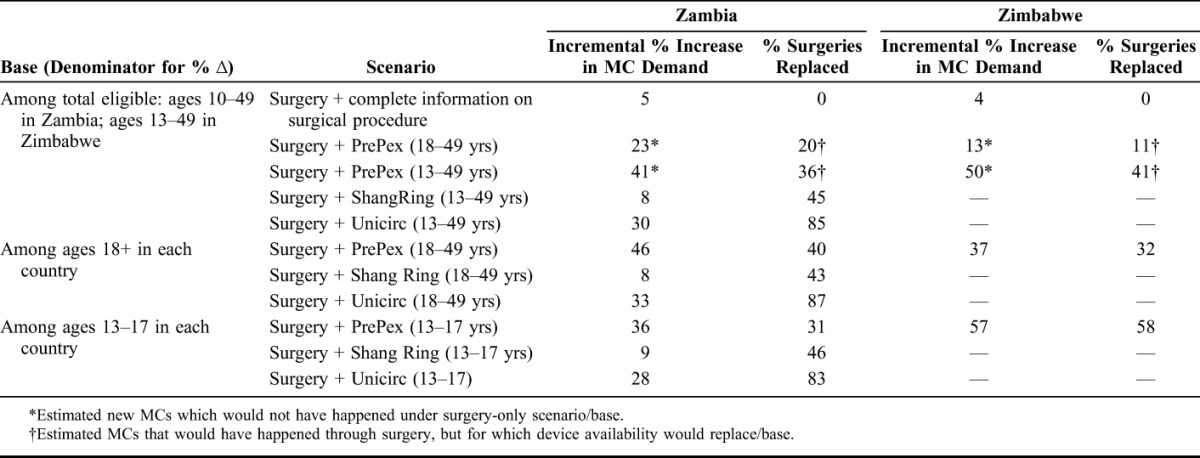
Incremental % Increase Demand vs. % Surgeries Replaced, by Device

**TABLE 2.**
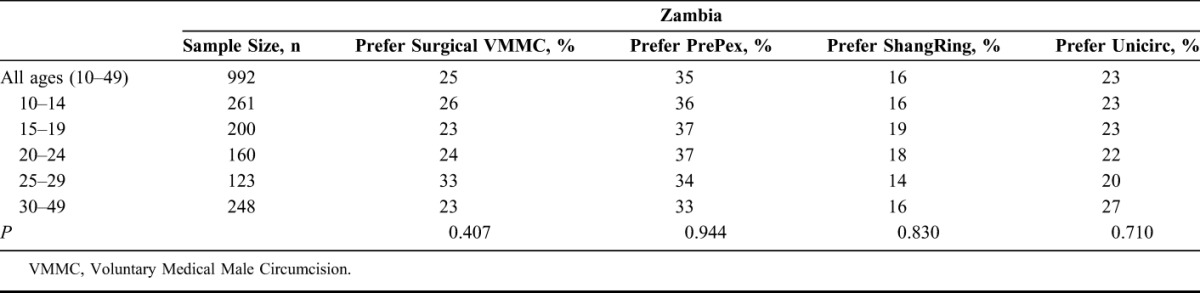
Preferred MC Procedure Type by Age for Zambia

**TABLE 3.**
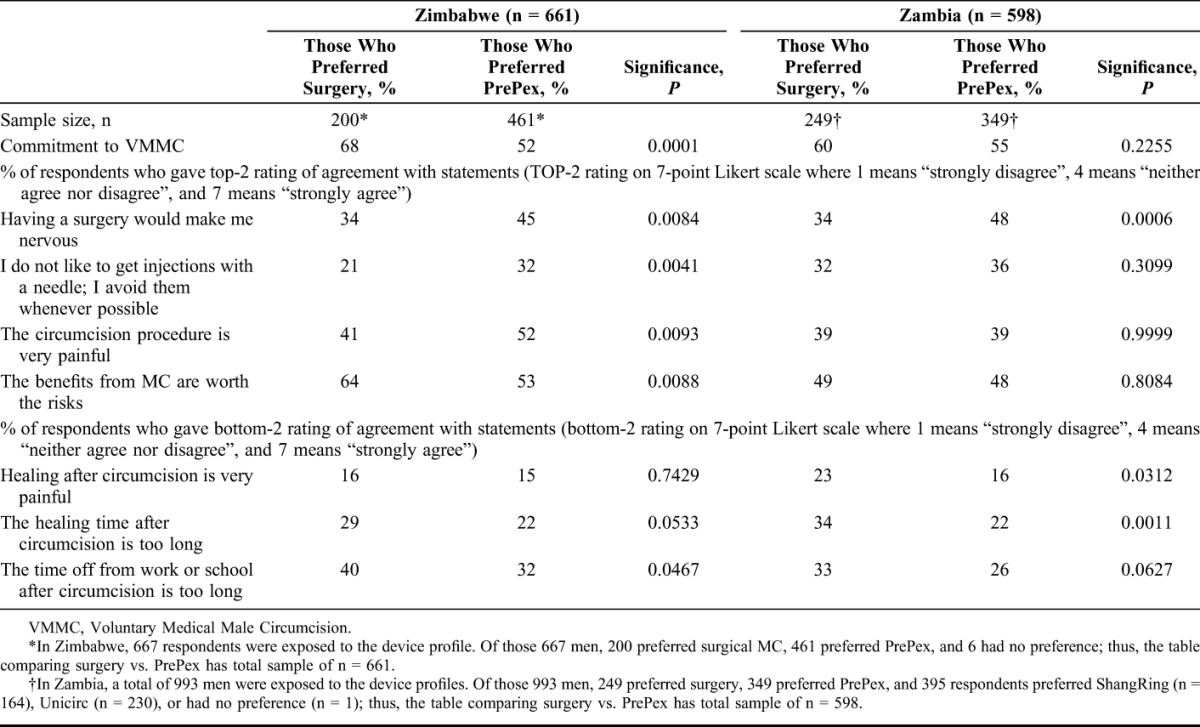
Perceptual Differences of MC by Procedure Method Preference (Boys and Men Preferring Surgery vs. Boys and Men Preferring PrePex)

### Potential for Devices in Scale-Up of MC, Zimbabwe

In Zimbabwe, complete information for the surgical procedure generated an estimated 4% incremental increase in modeled MC demand (Table 1). Modeled demand for PrePex resulted in an estimated 13% incremental increase in demand, while replacing 11% of surgeries for those aged 18–29. Including 13–17 year olds, PrePex could have created an estimated 50% incremental increase in MC demand, with an estimated 41% replacement of the 2014 surgical procedures. PrePex was the preferred MC option for all age groups with strongest support among men aged 15–19 (Table 4).

**TABLE 4.**
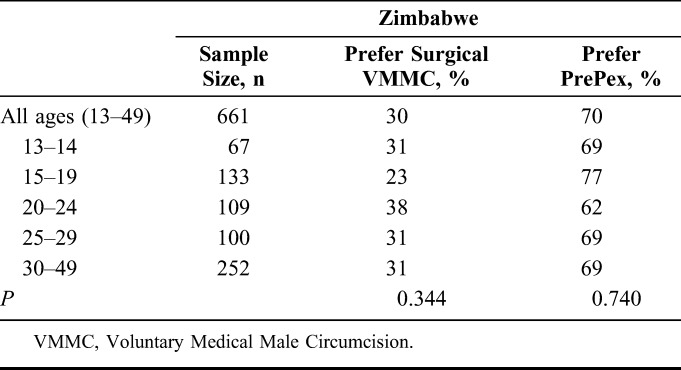
Preferred MC Procedure Type by Age for Zimbabwe

### Differential Procedure Preferences Among Men

In both countries, men who prefer surgery are more likely to have a relatively strong commitment to circumcision and communicate less fear of surgical procedures, injections, and anticipated pain of surgery than men who prefer PrePex, who are more likely to communicate greater concerns about surgery, injections, and fear of pain from the procedure (Table 2). Alternatively, the Unicirc and ShangRing procedures are preferred by many who would have opted for surgical MC, leading to heavy estimated replacement of the existing surgical service in Zambia of 85% and 45% for Unicirc and ShangRing, respectively. Responses and preferences and willingness to become circumcised after presentation of long profiles did not significantly differ to those given after the short profiles indicating that there was no evidence that there would be change of preference “at the clinic”, once full counseling information is provided.

### Limitations

The fundamental limitation of this model is that it presents a best-case scenario, which is likely unrealistic but can still be used as important input for decision making. Since the start of their MC programs, both countries achieved the highest levels of uptake for surgical MC in 2014. By assuming that awareness, promotion, and service for each device and surgical MC will continue at peak levels without a similar scale-up period for the selected device(s), we present an environment that is theoretical and unrealistic in the immediate term, but describes potential for the long term, after scale-up of devices. Because achieving this scenario would require high levels of program funding, training, and organizational efficiencies, achieving the modeled levels of demand for these devices would take time, stakeholder commitments, long-term investment, and robust program analysis.

## CONCLUSIONS

This research finds that introduction of devices can stimulate additional demand for MC, particularly among men and boys who are concerned about and fear the surgical procedure. PrePex has appeal to men who have these concerns and likely explains the highest incremental demand in each country for PrePex, with limited replacement of existing surgical service. If PrePex receives WHO prequalification for 13–17 year olds, this incremental demand increases. Although uptake for adolescents is already high, most are not yet circumcised in most of the priority countries. ShangRing and Unicirc appeal to boys and men who are also receptive to surgical MC, which, when coupled with the high medical eligibility rates for these devices, results in higher replacement rates for surgical MC relative to incremental demand. Unicirc is preferred to surgery and does generate substantial incremental demand, so it may provide for a long-term replacement of the surgical procedure, if cost of introduction and other factors affecting scale up are acceptable.

Integration of devices into existing programs is not seamless and will require consideration of many programmatic factors, including training of providers, quantification of need and distribution of multiple sizes, other costs of introduction, waste management, surgical backup, and referrals. Managing program services for 2 procedure types may be more complex to administer and coordinate and potentially costlier than managing a single service program. This could impact program efficiency and outweigh savings in delivery costs, at least during initial integration phases. Given the relatively high rate of ineligibility for PrePex among 13–17 year olds, the age group for which MC uptake is highest, surgery will always be a needed alternative with this approach, unless, for example, Unicirc proves to have equivalent medical eligibility rates as surgery and other factors warrant it replacing surgery. Within the current funding environment for MC programs globally, countries need robust evaluation of all factors, including potential impact on demand as modeled here, to make a data-driven decision on device introduction.
